# Nursing care of a child with cerebral palsy and multidrug-resistant bacterial pneumonia treated with bacteriophage therapy: a case report

**DOI:** 10.3389/fped.2026.1955901

**Published:** 2026-10-09

**Authors:** Lu Wang, Chunyan Lu, Huiwen Li, Dan Li

**Affiliations:** Department of Gastroenterology and Infectious Diseases, Shanghai Children's Hospital, Shanghai, China

**Keywords:** aerosol inhalation, bacteriophage therapy, case report, cerebral palsy, enteral nutrition, multidrug-resistant organisms, nursing

## Abstract

**Background:**

Children with cerebral palsy (CP) are at high risk of pulmonary infection with multidrug-resistant organisms (MDROs) owing to recurrent infections, prolonged hospitalization, and repeated antibiotic exposure. Bacteriophage (phage) therapy has emerged as a promising alternative for infections refractory to conventional antibiotics; however, its application in children with CP complicated by multidrug-resistant pneumonia has rarely been reported, and corresponding nursing experience remains scarce.

**Case presentation:**

This report describes the nursing care of a 2-year-and-4-month-old boy with CP, secondary epilepsy, and severe pneumonia caused by carbapenem-resistant *Acinetobacter baumannii* (CRAB), carbapenem-resistant *Klebsiella pneumoniae* (CRKP), and *Staphylococcus aureus* (discharge diagnosis: methicillin-resistant *S. aureus* infection), in whom conventional antibiotic therapy had failed. After comprehensive eligibility assessment, percutaneous endoscopic gastrostomy (PEG)-based nutritional optimization, and written informed consent, the child received a pre-planned 3-day course of nebulized phage cocktail therapy. Nursing care integrated standardized phage aerosol administration, staged enteral nutritional support, and family-centered health education. After treatment, *A. baumannii* was eradicated from bronchoalveolar lavage fluid, and subsequent *A. baumannii* isolates were susceptible to imipenem and meropenem (MIC ≤ 0.25 μg/mL, versus ≥16 μg/mL before therapy); CRKP was not re-isolated, and later *S. aureus* isolates were oxacillin-susceptible. Body temperature normalized, peripheral oxygen saturation remained stable without supplemental oxygen, and inflammatory markers decreased markedly. Body weight increased from 13.40 kg to 14.35 kg, and the nutritional risk score (STAMP) decreased from 5 (high risk) to 3. The child was discharged after 58 days of hospitalization.

**Conclusion:**

This case suggests that a structured nursing pathway embedding nebulized phage therapy is feasible and effective in medically complex children and may inform the care of similar pediatric cases.

## 1 Introduction

Cerebral palsy (CP) is a group of permanent disorders of movement and posture arising from non-progressive disturbances in the developing fetal or infant brain. Affected children frequently present with respiratory dysfunction, impaired airway clearance, and swallowing dysfunction, and are therefore highly susceptible to pulmonary infection (1). Owing to recurrent infections, prolonged hospitalization, and frequent antibiotic exposure, children with CP constitute a high-risk population for colonization and infection with multidrug-resistant organisms (MDROs) (2). According to the *Technical Guidelines for the Prevention and Control of Hospital Infections with Multidrug-Resistant Organisms (Trial)* issued by the former Ministry of Health of China, an MDRO is defined as a bacterium resistant to three or more classes of antimicrobial agents in clinical use (3). Such resistance is largely attributable to bacterial mutation and antimicrobial overuse. MDRO infections are characterized by clinical complexity, therapeutic difficulty, a propensity for colonization, and a protracted course. In children with CP who have dysphagia and severely limited mobility, effective treatment of such infections is particularly challenging—because of impaired airway clearance and recurrent microaspiration—and seriously compromises physical and mental health and quality of life.

The respiratory microbiota of children with CP is typically altered, with a decrease in commensal organisms and an enrichment of opportunistic pathogens (4). Carbapenem-resistant *Acinetobacter baumannii* (CRAB), carbapenem-resistant *Klebsiella pneumoniae* (CRKP), and methicillin-resistant *Staphylococcus aureus* (MRSA) are among the most frequently isolated pathogens (2). These organisms are designated priority pathogens by the World Health Organization (WHO) (5) and can impede antibiotic penetration through mechanisms such as biofilm formation, leading to failure of conventional therapy.

In recent years, bacteriophage (phage) therapy—including single-phage preparations, phage “cocktails,” and phage–antibiotic combinations—has attracted considerable attention as an emerging anti-infective strategy. Phages eliminate pathogenic bacteria through specific lytic activity, offering strong target specificity, a low propensity to induce resistance, and minimal disturbance of the normal flora (6). In a clinical summary of severe pulmonary infections caused by *A. baumannii*, the rate of clinical improvement or cure after phage therapy reached 71.4% (5/7), with no phage-related adverse reactions observed (7), lending preliminary support to the safety of this approach and offering a new option for the management of MDRO infections (8).

To our knowledge, phage therapy has not previously been reported in children with CP complicated by multidrug-resistant pneumonia; reported pediatric experience remains limited to a small number of cases in other conditions, such as cystic fibrosis (9, 36, 37), and nursing experience in this setting is correspondingly scarce. In April 2025, our department treated one such child with nebulized phage therapy. Following targeted interventions, the infection was effectively controlled and the overall rehabilitation outcome was satisfactory. This report summarizes the nursing practice to provide a reference for the care of similar cases.

## 2 Case presentation

### 2.1 Patient information

A boy aged 2 years and 4 months was admitted to our hospital on 4 April 2025 (Day 1) with a 14-day history of cough and wheezing, after ineffective treatment at another hospital. On examination, his temperature was 38.2 °C, pulse 143 beats/min, respiratory rate 35 breaths/min, blood pressure 103/57 mmHg, and peripheral oxygen saturation (SpO_2_) 86% on room air. Coarse breath sounds were heard bilaterally, with moist rales and wheezes. His height was 0.87 m and weight 13.40 kg.

### 2.2 Clinical findings and diagnostic assessment

Chest computed tomography (CT) demonstrated bilateral pulmonary infiltrates, and bronchoscopy revealed inflammation of the bronchial mucosa (endobronchitis). Gram-negative bacilli and Gram-positive cocci were identified in bronchoalveolar lavage fluid (BALF), and sputum culture yielded *A. baumannii*, *K. pneumoniae*, and *S. aureus*. The initial arterial blood gas analysis showed a PaO_2_ of 71.1 mmHg. Other laboratory tests revealed serum amyloid A 57.28 mg/L, platelets 551 × 10^9^/L, prothrombin time 13.5 s, serum potassium 3.20 mmol/L, serum calcium 1.17 mmol/L, and lactate 3.0 mmol/L. Albumin and total protein were marginally decreased; together with the hypermetabolic state and dysphagia associated with CP, these findings indicated a risk of occult malnutrition. Cranial imaging showed cerebral atrophy with basal ganglia encephalomalacia. At this admission, he was diagnosed with severe pneumonia with acute respiratory failure.

The child's past medical history included cerebral palsy, secondary epilepsy, hypoxic–ischemic encephalopathy, and cerebral atrophy following a previous near-drowning event, as diagnosed at another hospital; these conditions predated the acute presentation. Because of profound dysphagia, impaired airway clearance, and severely limited mobility, he had experienced recurrent episodes of wheezing and pulmonary infection, indicating chronic aspiration-related lung disease with habitual airway colonization. His Barthel Index score was 0, indicating complete dependence in activities of daily living and the need for comprehensive supportive treatment and long-term rehabilitation.

### 2.3 Therapeutic course

The overall clinical course and treatment timeline are summarized in Figure 1.

**Figure 1:**
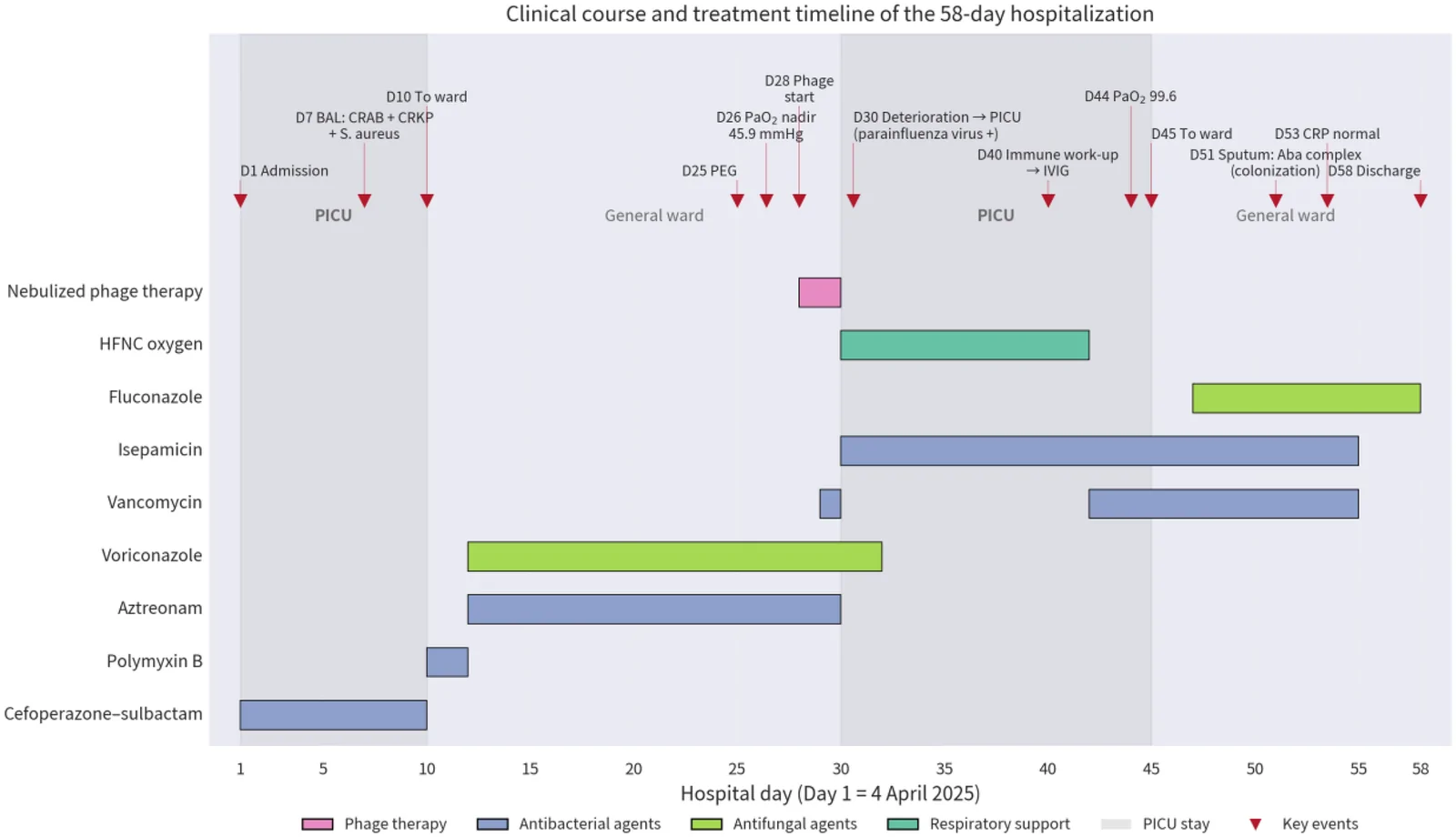
Clinical course and treatment timeline of the 58-day hospitalization. Horizontal bars indicate the administration periods of antimicrobial agents and respiratory support; shaded areas indicate PICU stays; red markers indicate key clinical events. Day 1 = 4 April 2025. BAL, bronchoalveolar lavage; CRAB, carbapenem-resistant *Acinetobacter baumannii*; CRKP, carbapenem-resistant *Klebsiella pneumoniae*; CRP, C-reactive protein; HFNC, high-flow nasal cannula; IVIG, intravenous immunoglobulin; PEG, percutaneous endoscopic gastrostomy.

#### 2.3.1 Initial empiric therapy and microbiological confirmation (days 1–10)

On admission (Day 1), the child was transferred to the pediatric intensive care unit (PICU) and received empiric anti-infective therapy (cefoperazone–sulbactam), together with anti-inflammatory, expectorant, and antiepileptic therapy and symptomatic support (Table 1). Because his respiratory status was initially too unstable to tolerate bronchoscopy, bronchoalveolar lavage was deferred until Day 7, when BALF culture and metagenomic next-generation sequencing confirmed mixed infection with *A. baumannii*, *K. pneumoniae*, and *S. aureus*. Sputum susceptibility testing (Day 6 and Day 14) identified carbapenem-resistant *A. baumannii* (CRAB; imipenem and meropenem MIC ≥ 16 μg/mL) and carbapenem-resistant *K. pneumoniae* (CRKP). Antibiotics were adjusted accordingly: polymyxin B (Days 10–12), followed by aztreonam combined with voriconazole (from Day 12; Table 1). Following this treatment, the child became afebrile with no convulsions under sedation, and his condition improved; on Day 10, he was transferred to our general ward for continued care on low-flow supplemental oxygen at 1 L/min (non-invasive ventilation was not required). However, despite this transient improvement, CRAB persisted in respiratory cultures (Day 14), and arterial blood gas analysis on Day 26 showed a nadir PaO_2_ of 45.9 mmHg (SpO_2_ 77.7%), indicating ongoing deterioration of gas exchange. The infection remained poorly controlled with available agents, and the overall condition continued to progress. The team therefore proceeded to evaluate the child's eligibility for phage therapy.

**Table 1:** Timeline of antimicrobial and phage therapy during the 58-day hospitalization.

Hospital day (date)	Agent	Dose, route, frequency	Notes
D1–D10 (Apr 4–13)	Cefoperazone–sulbactam (2:1)	0.5 g IV q8h	Empiric therapy
D10–D12 (Apr 13–15)	Polymyxin B	500,000 IU IV q12h	Targeting CRAB/CRKP
D12–D30 (Apr 15–May 3)	Aztreonam	0.45 g IV q8h	CRKP susceptible (MIC ≤ 1 μg/mL)
D12–D32 (Apr 15–May 5)^a^	Voriconazole	0.1 g IV q12h	Antifungal coverage
D28–D30 (May 1–3)	Phage cocktail (CP-p-AB-21016 + CP-p-AB-21006)	10^7^ PFU/mL, 5 mL per session, nebulized twice daily	Pre-planned 3-day course, completed
D29–D30 (May 2–3)	Vancomycin	0.2 g IV q8h	Gram-positive coverage
D30–D45 (May 3–18)	Isepamicin	150 mg IV qd	PICU period
D42–D45 (May 15–18)	Vancomycin	0.2 g IV q8h	PICU period
D45–D55 (May 18–28)	Isepamicin	135 mg IV qd	Consolidation in general ward
D45–D55 (May 18–28)	Vancomycin	0.18 g IV q8h	Consolidation in general ward
D47–D58 (May 20–31)^a^	Fluconazole	75 mg IV qd	Antifungal, continued to discharge

CRAB, carbapenem-resistant *Acinetobacter baumannii*; CRKP, carbapenem-resistant *Klebsiella pneumoniae*; IV, intravenous; qd, once daily; q8 h, every 8 h; q12 h, every 12 h. Carbapenems (imipenem/meropenem) were never administered during the entire hospitalization. Supportive agents not listed included methylprednisolone (15 mg IV q12 h/qd, PICU period), intravenous immunoglobulin, ambroxol, nebulized budesonide–ipratropium–salbutamol, and four antiepileptic agents (valproate 6.5 mL PO q12 h, levetiracetam 3 mL PO q12 h, diazepam 1.875 mg PO q6 h, clobazam 7.5 mg PO qd).

a Voriconazole and fluconazole were each briefly interrupted during the course; the dates shown indicate the overall treatment windows.

#### 2.3.2 Nutritional optimization before phage therapy (days 10–28)

To establish a long-term enteral nutrition route for the child's dysphagia and malnutrition—and thereby improve his tolerance of subsequent phage therapy—percutaneous endoscopic gastrostomy (PEG) was performed on Day 25; gastroscopy during the same procedure revealed esophagitis, carditis, chronic superficial gastritis with erosion, and erosive duodenitis. With standardized enteral feeding thereafter, his nutritional status gradually improved and body weight and albumin stabilized. After the family provided written informed consent, the eligibility criteria for phage therapy were met.

#### 2.3.3 Nebulized phage therapy (days 28–30)

On Day 28, nebulized phage therapy was initiated at a frequency of twice daily for 3 consecutive days; the 3-day course was the pre-planned regimen and was completed in full. Before each session, thorough airway suctioning was performed; during administration, vital signs were closely monitored for allergic reactions, bronchospasm, and endotoxin-release reactions; and suctioning was withheld for 20 min after each session to preserve drug concentration in the lungs. Vancomycin was added on Day 29 for Gram-positive coverage.

#### 2.3.4 Post-treatment deterioration and PICU course (days 29–45)

On Day 29, C-reactive protein (CRP) rose sharply to 51.21 mg/L, and on Day 30—the final day of the phage course—the child developed recurrent fever, tachypnea, and increased cough and sputum production, consistent with acute exacerbation of pulmonary infection, and his condition became critical. Serum endotoxin was <5.00 EU/(kg·h), excluding a phage-related endotoxin-release reaction, and respiratory pathogen testing detected parainfluenza virus; the deterioration was therefore attributed to progression of the underlying disease with superimposed infection rather than to phage therapy. He was transferred back to the PICU on Day 30 for intensive care.

During the 15-day PICU stay (Days 30–45), inflammatory markers peaked on Days 30–31 (CRP 88.05 mg/L, interleukin-6 870.91 pg/mL, serum amyloid A 213.35 mg/L) and then declined gradually. The child received high-flow nasal cannula (HFNC) oxygen therapy from Day 30 to Day 42, without escalation to invasive mechanical ventilation. Antimicrobial therapy comprised isepamicin (throughout the PICU stay), vancomycin (Days 42–45), and voriconazole (Days 30–32), together with intravenous methylprednisolone, nebulized budesonide–ipratropium–salbutamol, and intravenous ambroxol (Table 1). Repeat bronchoscopy was performed on Day 36. Immunological work-up on Day 40 showed decreased serum IgM (0.13 g/L), CD3^+^ T cells 51.79%, CD4^+^ T cells 22.94%, and a CD4/CD8 ratio of 0.87, confirming impaired cellular and humoral immunity; intravenous immunoglobulin was administered as immune support. Secondary epilepsy remained difficult to control and required four concurrent antiepileptic agents (valproate, levetiracetam, diazepam, and clobazam). Nursing care during this period focused on HFNC management with close monitoring of oxygenation and work of breathing, airway clearance, seizure surveillance and safety protection, and meticulous PEG tube care. With this treatment, his condition stabilized, oxygenation recovered (PaO_2_ 99.6 mmHg on Day 44), and on Day 45 he returned to our ward for consolidation therapy with isepamicin, vancomycin, and fluconazole (Table 1).

### 2.4 Outcomes

Ultimately, the child's temperature normalized and SpO_2_ remained stable without supplemental oxygen. The three target organisms evolved as follows. (1) *A. baumannii*: repeat BALF culture after phage therapy no longer yielded the organism. The pre-treatment CRAB isolate (Day 14) was resistant to imipenem and meropenem (MIC ≥ 16 μg/mL), whereas *A. baumannii* complex isolated from sputum on Days 29 and 33 was fully susceptible to both agents (MIC ≤ 0.25 μg/mL), indicating loss of the carbapenem-resistant phenotype after phage therapy. A late sputum culture (Day 51) still grew *A. baumannii* complex together with *Candida albicans*; given the child's sustained clinical improvement and the radiographic absorption of the pulmonary infiltrates, this finding was interpreted as airway colonization rather than active infection, and no escalation of antimicrobial therapy was required. (2) *K. pneumoniae*: CRKP was last isolated on Day 14 and was not detected in any subsequent respiratory culture; the isolate had been susceptible to aztreonam (MIC ≤ 1 μg/mL) and amikacin, which were included in the treatment regimen. (3) *S. aureus*: the organism was isolated from sputum on Days 29 and 33, but these isolates were oxacillin-susceptible (MIC ≤ 0.25 μg/mL); two MRSA colonization screening swabs were negative, although the discharge diagnosis retained “MRSA infection” on the basis of the initial BALF finding. Inflammatory markers decreased substantially (CRP < 10 mg/L and serum amyloid A within the normal range by Day 53), and follow-up chest CT showed marked absorption of the bilateral pulmonary infiltrates. The child improved and was discharged on Day 58 of hospitalization. At approximately 6 months after discharge, the child remained clinically stable on gastrostomy feeding and was electively readmitted for scheduled PEG tube replacement, with no recurrence of multidrug-resistant organism infection.

## 3 Nursing interventions

### 3.1 Precise management of multidrug-resistant infection

#### 3.1.1 Assessment and preparation before phage therapy

BALF culture identified CRAB, CRKP, and *S. aureus*—the first two listed among the WHO bacterial priority pathogens (5). Sequential conventional regimens—including cefoperazone–sulbactam, polymyxin B, and aztreonam—had failed to eradicate CRAB, and the child's condition continued to progress. With reference to the *Expert Consensus on Clinical Application Management of Phage Therapy (2024 Edition)* (10), the team conducted a comprehensive eligibility assessment: (1) sputum culture and antimicrobial susceptibility testing confirmed MDRO infection (3); (2) clinical symptoms had failed to improve, or had worsened, after more than 72 h of conventional antibiotic therapy; (3) *in vitro* testing confirmed that the isolated pathogens were susceptible to the laboratory-prepared phage preparations; and (4) the family provided written informed consent. The child therefore had a clear indication for phage therapy and met the ethical requirements for enrollment.

Phage therapy exploits the specific lytic activity of bacteriophages against pathogenic bacteria. Routes currently explored or applied include intravenous injection, oral administration, topical application, intracavitary instillation, and inhalation (11). Rational selection of the administration route determines whether phages reach the infection site at an adequate concentration and is a key determinant of therapeutic success (11). In this child, long-term treatment had made venipuncture difficult, and intravenous infusion was painful and carried risks of failed administration and extravasation. Dysphagia secondary to CP precluded reliable oral dosing and posed an aspiration risk. Because the infection was predominantly pulmonary and required drug delivery directly to the airway lesions, aerosol inhalation was selected as the most appropriate route: it is non-invasive, well tolerated, and allows preferential deposition of phage preparations at the site of pulmonary infection (12), thereby improving efficacy while reducing the risks of venous injury and aspiration.

Nevertheless, several barriers delayed initiation of phage therapy until Day 28. First, the child's poor baseline condition, high early nutritional risk, and limited physiological tolerance necessitated prior optimization of his general status. Second, as phage therapy is a novel technology, the family had limited knowledge and understandable concerns, and health education and the informed-consent process required a prolonged communication cycle. Third, the team had limited experience in predicting the trajectory of MDRO infection and the risk of endotoxin release, which contributed to the relatively late intervention. After systematic nutritional support and targeted education on the safety and efficacy of phage therapy, the family provided informed consent, and—with the child's nutritional status meeting requirements—phage therapy was formally initiated on Day 28.

To ensure drug potency and treatment safety, operators strictly observed aseptic principles (10) and standardized preparation management and airway pre-treatment. Phage lysate preparations were stored refrigerated at 4 °C; different preparations were stored separately, and storage temperature was monitored regularly to preserve phage activity (13). Before use, the batch number, expiry date, and appearance of each preparation were verified to confirm clarity without turbidity, precipitation, or cold-chain interruption (13). The two patient-specific preparations (CP-p-AB-21016 and CP-p-AB-21006) were independently double-checked before aseptic compounding. Given the child's CP and limited ability to cooperate, a face-mask nebulizer was selected (14). Before each session, the team prepared negative-pressure suction and emergency equipment and monitored vital signs throughout, remaining vigilant for endotoxin-release reactions, allergy, bronchospasm, and shock (15). Immediately before nebulization, the child's back was percussed and airway secretions were cleared to reduce the physical barrier posed by sputum and to ensure delivery of phage preparations to the deep airways (16).

#### 3.1.2 Nursing care during phage administration

Phage nebulization generates aerosols that may provoke stress reactions in children with severe underlying disease (10) and increase the risks of environmental contamination and occupational exposure. Accordingly, nursing staff wore isolation gowns, N95 respirators, and gloves during each session to block transmission of pathogens and phage particles and to prevent exposure and cross-infection (17). Feeding was withheld for 30 min before nebulization, and sessions were conducted while the child was quiet or asleep. Because the child could not sit upright, the head of the bed was elevated by 30° to achieve a comfortable semi-recumbent position (14). The mask was held snugly against the face, and soothing and gentle touch were used to minimize crying and choking, thereby avoiding drug leakage and airway irritation and ensuring effective deposition of the phage preparation at the pulmonary lesions (18).

A CRAB-targeted phage cocktail comprising preparations CP-p-AB-21016 and CP-p-AB-21006 was formulated to a final titer of 10^7^ PFU/mL for nebulization (10). The preparation was administered via face-mask inhalation twice daily, 20 min per session, using 5 mL of solution per session. Operators strictly controlled nebulization parameters: the oxygen flow rate was initially set at 1.5 L/min and gradually increased to 4 L/min according to the child's tolerance and ventilatory requirements, to achieve an aerosol particle size appropriate for therapeutic efficacy (19). In addition to monitoring for allergy and bronchospasm, the team remained alert to transient fever or inflammatory exacerbation caused by endotoxin release from phage-mediated bacterial lysis (20). If tachypnea or desaturation occurred, the child's breathing was guided and the oxygen flow increased to 4 L/min; if significant discomfort or a change in condition was observed, nebulization was suspended immediately, oxygen and other symptomatic support were provided, and the physician was notified promptly (14).

#### 3.1.3 Post-treatment care and evaluation of therapeutic response

Post-treatment nursing is essential to preserve efficacy and comfort. Suctioning was strictly withheld for 20 min after each session (13) to avoid removing the deposited drug and to reduce the risk of airway irritation–induced bronchospasm. After nebulization, staff assisted the family in cleaning the child's mouth, nose, and facial skin with warm water to relieve pharyngeal discomfort and remove drug residue (21), and then kept the child calm to avoid vigorous coughing and airway irritation, allowing the drug to deposit uniformly and remain in the lungs (18). During this period, vital signs were monitored dynamically and rigorously, and cough frequency as well as the volume, viscosity, and color of sputum were observed to assess airway patency and therapeutic response (17). After each session, the instruments and equipment used were disinfected: equipment surfaces were wiped with intermediate- or high-level disinfectant solutions or wipes, or, as ordered by the physicians, sprayed with bacteriophage preparations targeting the relevant bacteria for decontamination (17); disposable consumables were discarded as infectious medical waste. Finally, the treatment time, phage dose, and vital-sign changes at each stage were documented completely and accurately.

Phages can modulate the resistance mechanisms of MDROs, restore antibiotic susceptibility, and act synergistically with antibiotics to enhance anti-infective effects (22). After treatment, the child's cough diminished, sputum became thinner and easier to expectorate, vital signs remained stable, and mental status and appetite improved markedly. Repeat BALF culture after treatment no longer yielded *A. baumannii*, and post-treatment *A. baumannii* complex isolates were susceptible to imipenem and meropenem (MIC ≤ 0.25 μg/mL, versus ≥ 16 μg/mL in the pre-treatment CRAB isolate); a late sputum culture remained positive for *A. baumannii* complex, which was interpreted as colonization in the context of sustained clinical and radiographic improvement; C-reactive protein fell below 10 mg/L, pulmonary moist rales decreased, and chest CT showed absorption of the bilateral pulmonary infiltrates.

### 3.2 Nutritional support to improve nutritional status

#### 3.2.1 Multidimensional nutritional screening and assessment

Malnutrition and hypoalbuminemia are independent risk factors for MDRO infection, and systematic nutritional intervention is therefore required during treatment to improve the host immune microenvironment (23, 24). Nutritional support contributes not only to recovery but also to immune reconstitution (25), and accurate nutritional risk assessment is the prerequisite for an effective intervention plan (26). In this case, the Screening Tool for the Assessment of Malnutrition in Paediatrics (STAMP) was used (27). The child scored 5 points, meeting the criteria for high nutritional risk (a STAMP score of ≥4 indicates high risk). His albumin was at the lower limit of normal, and the hypermetabolic state of CP, feeding difficulty, and dysphagia further aggravated the nutritional imbalance. An individualized nutritional support plan was therefore formulated, aimed at improving the immune microenvironment, enhancing resistance, and reducing the risk of secondary infection.

#### 3.2.2 Escalation of the nutritional support route and tube care

Given the high nutritional risk, dysphagia, and inadequate oral intake, conventional feeding could not meet the child's physiological needs. For children with normal gastrointestinal function who require long-term (>4–6 weeks) enteral nutrition, PEG is the clinical gold standard for establishing a safe and stable feeding route (26). Postoperatively, care focused on preventing stoma infection and unplanned tube removal, through daily inspection of the peristomal skin, routine disinfection and dressing changes, and secure fixation of the tube (28).

#### 3.2.3 Enteral nutrition intervention and outcomes

For such children, 111 kcal/kg may be used as an estimate of energy requirements (26, 29); accordingly, a high-energy-density enteral formula was selected to improve nutritional status. With standardized care and nutritional support, nutritional risk can be markedly reduced (30); however, given this child's profound dysphagia, long-term gastrostomy feeding was anticipated rather than weaning to full oral intake. Nutritional support followed a stepwise principle: the enteral feeding pump was started at a continuous rate of 10–20 mL/h and increased by 5–10 mL/h every 12–24 h until the target energy intake was reached. For adjunctive management of the child's difficult-to-control secondary epilepsy, which required four concurrent antiepileptic agents, the enteral formula was converted to a ketogenic formulation with a ratio of 3:1 or 4:1 as prescribed by the treating physicians, and the tube was flushed thoroughly with water at 35–40 °C to prevent blockage by the high-fat formula (31).

Because enteral nutrition is a dynamic process, a rigorous monitoring and evaluation system was established to ensure effectiveness and safety. Steady weight gain served as the primary outcome indicator: body weight was measured twice weekly and plotted on a growth curve; gastrointestinal tolerance was closely observed to guide the rate of escalation; and blood counts, liver and kidney function, and electrolytes were reviewed regularly, with particular attention to the recovery of nutritional indices such as albumin and total protein (32).

After systematic implementation of the enteral nutrition plan, the child's total protein increased from 61.34 g/L to 69.40 g/L, indicating significant nutritional improvement. Body weight increased continuously (with an interruption during the PICU stay from Day 30 to Day 45), rising from 13.40 kg at admission to 14.35 kg at discharge. The nutritional risk decreased substantially, with the STAMP score falling from 5 (high risk) to 3 (moderate risk), confirming a definite effect of the enteral nutrition intervention.

### 3.3 Family-centered health education to strengthen home management capacity

After completion of phage therapy, the child entered the home rehabilitation stage. Consensus recommendations require follow-up for at least 4 weeks after the final phage administration and, where feasible, long-term follow-up for 1 year or more, with regular outpatient review and dynamic monitoring of pathogens, inflammatory markers, and growth and development, to assess long-term efficacy and the risk of recurrence (10). Before discharge, family caregivers received structured training in the areas most relevant to this child's safety: daily PEG stoma care and tube flushing to prevent blockage and infection (33); seizure first aid, including lateral positioning and airway protection (34); and infection prevention, including hand hygiene and home pulmonary physiotherapy (35). A follow-up file was established, and regular telephone follow-up was arranged to support home care.

## 4 Discussion

This report describes the nursing care of a child with CP and multidrug-resistant bacterial pneumonia who received nebulized phage therapy. By standardizing the nursing process for phage aerosol administration, rationally adjusting the enteral nutrition regimen during treatment, and providing targeted caregiver education, we developed an integrated nursing model suited to medically complex children.

Several features of this case warrant emphasis. First, the choice of the aerosol route balanced efficacy against the child's specific constraints—difficult venous access, dysphagia-related aspiration risk, and a predominantly pulmonary infection—allowing direct delivery of phage preparations to airway lesions while remaining non-invasive (11, 12). Second, the experience highlights the importance of host optimization before phage therapy: the delay in initiation was largely attributable to poor baseline nutritional status and limited physiological tolerance, and staged nutritional support via PEG proved to be both a prerequisite for safe phage administration and an independent contributor to recovery, as reflected in the improvement of total protein, body weight, and STAMP score. Third, the transient post-treatment deterioration underscores the need for vigilance regarding endotoxin-release reactions and the value of objective differentiation—through endotoxin monitoring—between phage-related adverse events and progression of the underlying disease (20). Finally, after phage therapy the *A. baumannii* complex isolates recovered from sputum were fully susceptible to carbapenems (MIC ≤ 0.25 μg/mL), in contrast to the pre-treatment CRAB isolate (MIC ≥ 16 μg/mL). Because strain typing was not performed, we cannot distinguish between phage-mediated resensitization of the original CRAB clone and eradication of the resistant clone with subsequent colonization by susceptible strains; notably, carbapenems were never administered during this admission, excluding selection pressure from these agents. This observation is consistent with reported mechanisms by which phages resensitize MDROs to antibiotics and supports phage–antibiotic synergy as a therapeutic concept (22). It is also noteworthy that a late sputum culture remained positive for *A. baumannii* complex despite clearance from BALF, underscoring the importance of distinguishing airway colonization from active infection when interpreting post-treatment cultures.

This single-case experience has inherent limitations. Conclusions regarding efficacy cannot be generalized; although no recurrence was observed at the 6-month follow-up, longer-term surveillance remains warranted; and several elements of the nursing pathway—such as the prediction of infection trajectory and endotoxin-release risk—were refined only through this experience. Nevertheless, the case suggests that nebulized phage therapy, when embedded in a structured nursing framework, is clinically feasible and safe for children with multidrug-resistant pneumonia and can effectively control infection and improve prognosis.

## 5 Conclusion

This case describes, to our knowledge, the first reported application of nebulized phage therapy in a child with CP complicated by multidrug-resistant bacterial pneumonia, encompassing both the clinical application and trajectory of this novel therapy and the practical nursing experience that supported it. A care model integrating standardized phage aerosol administration, staged PEG-based nutritional support, and family-centered health education was associated with clearance of CRAB from the lower airways (BALF), loss of the carbapenem-resistant phenotype in subsequent *A. baumannii* isolates, resolution of infection, improved nutritional status, and successful discharge. Beyond nursing, this case provides clinical evidence for the feasibility and safety of nebulized phage therapy in medically complex children, and its trajectory—from eligibility assessment and host optimization to administration, adverse-event differentiation, and microbiological follow-up—may inform multidisciplinary teams considering phage therapy for similar patients. In the future, the entire phage-therapy process—preparation storage, compounding, inhalation technique, efficacy monitoring, and complication prevention—should be systematized into graded, standardized protocols. Continued accumulation of pediatric evidence may gradually yield a unified, standardized pathway, providing a stronger foundation for the safe clinical translation of phage therapy in refractory pediatric MDRO infections.

## Data Availability

The raw data supporting the conclusions of this article will be made available by the authors, without undue reservation.
